# Validation of the 33-Item Hypomania Checklist-External Assessment in Screening Adolescents for Bipolar Disorder

**DOI:** 10.3389/fpsyt.2022.897357

**Published:** 2022-06-15

**Authors:** Xu Chen, Hong Cai, Wei Bai, Sha Sha, Teris Cheung, Gabor S. Ungvari, Yuan Feng, Yu-Tao Xiang, Jules Angst

**Affiliations:** ^1^The National Clinical Research Center for Mental Disorders and Beijing Key Laboratory of Mental Disorders, Beijing Anding Hospital and The Advanced Innovation Center for Human Brain Protection, Capital Medical University, Beijing, China; ^2^Unit of Psychiatry, Department of Public Health and Medicinal Administration, Faculty of Health Sciences, Institute of Translational Medicine, University of Macau, Taipa, Macao SAR, China; ^3^Centre for Cognitive and Brain Sciences, University of Macau, Taipa, Macao SAR, China; ^4^Institute of Advanced Studies in Humanities and Social Sciences, University of Macau, Taipa, Macao SAR, China; ^5^School of Nursing, Hong Kong Polytechnic University, Kowloon, Hong Kong SAR, China; ^6^Division of Psychiatry, School of Medicine, University of Western Australia, Perth, WA, Australia; ^7^Section of Psychiatry University of Notre Dame Australia, Fremantle, WA, Australia; ^8^Zurich University Psychiatric Hospital, Zurich, Switzerland

**Keywords:** validation, HCL-33-EA, major depressive disorder, bipolar disorder, adolescents

## Abstract

**Background:**

Bipolar disorder (BD) is often misdiagnosed, leading to poor treatment outcomes. Thus, accurate assessment of BD is of great importance, including in BD adolescents. The aim of the study was to explore the psychometric properties of the 33-item Hypomania Checklist-External Assessment (HCL-33-EA) in depressed adolescents.

**Methods:**

The study was conducted between March and November 2020 in Beijing, China. Depressed adolescents aged between 13 and 17 years (*N* = 260) with BD (*N* = 147) or major depressive disorder (MDD) (*N* = 113) diagnosed according to the International Classification of Diseases, Tenth Revision (ICD-10) were recruited. Patients’ hypomanic symptoms were assessed by their carers using the HCL-33-EA.

**Results:**

The HCL-33-EA showed high internal consistency (Cronbach’s alpha = 0.82) with two factorial dimensions. The Receiver Operating Characteristic (ROC) curve analysis revealed an area under the ROC curve (AUC) value of 0.61 (95% confidence interval (CI): 0.54–0.67). The optimal cut-off score of 7 generated the best combination of sensitivity (0.81) and specificity (0.37) for discriminating between adolescents with BD and MDD.

**Conclusion:**

The HCL-33-EA, with a two-factor structure, seems to be a useful tool for screening for BD in depressed adolescents. However, the high sensitivity and low specificity of the HCL-33-EA at the optimal cut-off value of 7 indicate that the HCL-33-EA needs to be further refined for young patients.

## Introduction

Bipolar disorder (BD) is characterized by extreme changes in mood, with alternating episodes of mania, hypomania, depression, or a mixed mood state (1). For at least half of those who develop BD, the illness may start in childhood and lead to dysfunction in areas such as education, employment, independent living, and marriage. Individuals whose depressive episodes are more severe or with longer duration or frequent recurrences have a higher risk of substance use (2, 3). The overall prevalence of BD in children and adolescents is estimated to be 0.05% globally (4). Adolescents with BD show increased energy, irritability, labile mood, distractibility, euphoria/elated mood, hyperactivity, pressured speech, racing thoughts, flight of ideas, poor judgment, grandiosity, and decreased sleep (5, 6). Failure to accurately identify adolescents’ atypical presentation can result in misdiagnosis and inappropriate treatment of BD with detrimental clinical consequences (3, 7) including prescription of antidepressants (8). The accurate assessment of their illness is thus of paramount importance for adolescents with BD. It is therefore pivotal for clinicians to carefully screen and monitor depressed adolescents if they show early signs of mood symptoms, especially if they exhibit sleep disturbances, anxiety attacks, and irritability.

The self-report 33-item Hypomania Checklist (HCL-33) is a modified version of the 32-item Hypomania Checklist (HCL-32) (9). It has been validated for screening for BD in depressed adults and adolescents (10, 11). The 33-item Hypomania Checklist-External Assessment (HCL-33-EA) was developed for rating patients with mood disorders by their carers (12) and its Chinese version has been validated in adults for screening for BD in depressed patients with acceptable psychometric properties (10). It could be an effective screening tool for patients’ carers, enabling the identification of hypomanic symptoms. Since the HCL-33-EA has not yet been validated in adolescents, the aim of the study was to explore the psychometric properties of the HCL-33-EA in depressed adolescents in China.

## Materials and Methods

### Study Sample and Site

The study was conducted between March and November 2020 in the Department of Child Psychiatry, Beijing Anding Hospital of Capital Medical University, China. Patients and their carers attending the outpatient clinics were invited to participate in the study. To be eligible, participants were (1) aged between 13 and 17 years old; (2) diagnosed with BD (ICD-10 code: F31) or major depressive disorder (MDD; ICD-10 code: F32.0-F32.5; F32.9; F33) according to the International Classification of Diseases, Tenth Revision (ICD-10) (13) by a consensus of two senior psychiatrists; (3) scored 7 or higher on the 17-item Hamilton Depression Rating Scale (HAMD) (14, 15) at the routine screening at the outpatient department prior to their consultation; (4) able to understand the aim and contents of the assessment and provide verbal informed consent, whilst their carers gave written informed consent. Persons with cognitive impairment were excluded. Inclusion criteria for carers were: (1) patients’ parents or legal guardians; (2) no current major psychiatric disorders and/or cognitive impairment; (3) able to understand the aim and contents of the assessment. The study protocol was approved by the Medical Ethics Committee of Beijing Anding Hospital.

### Instrument and Data Collection

Eligible participants were interviewed by one of two research psychiatrists. Patients’ demographic and clinical characteristics were collected through a review of their medical records and confirmed in the interview. Caregivers’ demographic characteristics were collected in the interview by two research psychiatrists. The Chinese version of the HCL-33-EA (12) was used to assess patients’ hypomanic symptoms by their carers. Each item of the HCL-33-EA has a dichotomous response option (yes/no); the total score of the HCL-33-EA is the sum of all items with a “yes” response. The values of the HCL-33-EA items were dichotomized as “0” or “1,” with the total score ranging from 0 to 33.

### Statistical Analyses

The Statistical Package for Social Sciences (SPSS), Version 26.0, was used for all analyses. The HCL-33-EA total score and the frequencies of positive responses to each HCL-33-EA item by the MDD and BD patient carer groups were compared. The normal distribution of continuous variables was examined by p-p plot. Normally distributed continuous variables were compared with the two independent sample *t*-test, while skewed continuous variables were compared with the Mann-Whitney test. Categorical variables (e.g., frequencies of positive responses to each HCL-33-EA item) were compared using the chi-square test. The factor structure of the HCL-33-EA was explored by principal component factor analysis. Factors with Eigenvalues larger than 1 were identified and the final number of factors was determined by clinical consideration; items were allocated to a factor if their loading values were > 0.4, as recommended (9). The internal consistency was tested with Cronbach’s alpha with a value of > 0.7 indicating acceptable reliability (16). The split half reliability was calculated using the Spearman-Brown coefficient with the coefficient value of > 0.7 signaling acceptable reliability (16). The Receiver Operating Characteristic (ROC) curve analysis was conducted to estimate the sensitivity and specificity at each cut-off value of the HCL-33-EA. The area under the ROC curve (AUC) was calculated to test the ability of the HCL-33-EA to discriminate between MDD and BD. AUC > 0.6 represented acceptable discrimination (17). The Youden’s index (maximum value by adding up sensitivity and specificity at each cut-off value) was adopted to identify the optimal cut-off value (18). The significance level was set at *p* < 0.05 (2-tailed).

## Results

### Demographic Characteristics of Patients and Their Carers

A total of 260 patients were recruited to the study; 113 were diagnosed with MDD and 147 with BD. Patients’ demographic and clinical characteristic are presented in Table 1. The carers’ demographic information is shown in Table 2.

**TABLE 1:** Basic demographic and clinical characteristics of adolescents diagnosed with MDD or BD.

Variables	Whole sample (*n* = 260)		MDD (*n* = 113)		BD (*n* = 147)		MDD vs. BD	
	Mean	SD	Mean	SD	Mean	SD	Z*	*P*
Age (years)	15.42	1.62	15.30	1.51	15.51	1.70	−1.015	0.310
Education level (years)	9.76	1.94	9.45	1.93	10.0	1.93	−1.848	0.065
Age at first onset (years)	14,33	4.21	14.12	5.40	14.41	3.01	−1.170	0.242
Total number of typical depressive or manic episodes	1.20	0.85	1.24	1.18	1.17	0.46	−0.294	0.768

	***N***	**%**	***N***	**%**	***N***	**%**	**χ^2^**	***P***

Male gender	59	22.7	31	27.4	28	19.0	2.561	0.110

*BD, bipolar disorder; MDD, major depressive disorder; SD, standard deviation.*

*Mann-Whitney U-test.

**TABLE 2:** Demographic information of participants’ carers.

Variables	Whole sample (*n* = 260)		MDD (*n* = 113)		BD (*n* = 147)		MDD vs. BD	
	Mean	*SD*	Mean	*SD*	Mean	*SD*	χ^2^	*P*
Age (years)	43.09	5.28	43.09	6.42	43.10	4.22	−0.938	0.348
Education level (years)	13.71	3.02	14.02	3.14	13.74	2.91	−1.864	0.062

	***N***	**%**	***N***	**%**	***N***	**%**	Z*	***P***

Male gender	58	22.3	27	23.9	31	21.1	0.290	0.590
Married	244	93.8	108	95.6	136	93.5	1.578	0.209
Employed	199	76.5	86	76.1	113	76.9	1.035	0.309

*BD, bipolar disorder; MDD, major depressive disorder; SD, standard deviation.*

*Mann-Whitney U-test

### Comparison Between the 33-Item Hypomania Checklist-External Assessment Scores of the Major Depressive Disorder and Bipolar Disorder Patient Groups

MDD patients had significantly lower total scores on the HCL-33-EA than BD patients [mean (*M*) = 9.26, standard deviation (*SD*) = 5.44] vs. (*M* = 11.26, *SD* = 5.44), *Z* = −2.914, *p* = 0.004). The frequency of responses to items 1, 2, 8, 10, 11, 13, and 30 also differed significantly between the two groups (Table 3).

**TABLE 3:** HCL-33-EA—Percentage of positive responses by carers of depressed adolescents with MDD or BD.

HCL-33	Frequency of positive response (%)	
	MDD	BD
He/she needs less sleep	23.0	36.1*
He/she is more energetic and more active	52.2	66.7*
He/she is more self-confident	43.4	52.4
He/she enjoys his/her work more	32.7	36.7
He/she is more sociable (makes more phone calls, goes out more)	38.1	38.8
He/she wants to travel and/or does not travel more	41.6	44.9
He/she tends to drive faster or take more risks when driving	8.0	8.8
He/she spends more money/too much money	23.9	37.4*
He/she takes more risks in daily life (in his/her work and/or other activities)	8.0	11.6
He/she is physically more active (sport, etc.)	15.9	27.9*
He/she plans more activities or projects	32.7	47.6*
He/she has more ideas, is more creative	45.1	51.7
He/she is less shy or inhibited	24.8	38.1*
He/she wears more colorful and more extravagant clothes/make-up	15.9	22.4
He/she wants to meet or actually does meet more people	18.6	22.4
He/she is more interested in sex and/or is more sexually active	5.3	9.5
He/she talks more	53.1	61.2
He/she thinks faster	54.0	61.2
He/she makes more jokes or puns when talking	45.1	56.5
He/she is more easily distracted	40.7	36.7
He/she engages in lots of new things	35.4	42.9
His/her thoughts jump from topic to topic	30.1	32.0
He/she does things more quickly and/or more easily	37.2	49.0
He/she is more impatient and/or gets irritable more easily	51.3	53.7
He/she can be exhausting or irritating for others	36.6	37.4
He/she gets into more quarrels	23.9	27.2
His/her mood is higher, more optimistic	41.6	51.0
He/she drinks more coffee	8.0	4.8
He/she smokes more cigarettes	2.7	2.7
He/she drinks more alcohol	0.9	5.4*
He/she takes more drugs (sedatives, anxiolytics, stimulants)	4.4	5.4
He/she games or gambles more	15.0	22.4
He/she eats more or binges more	15.0	21.8

*BD, bipolar disorder; MDD, major depressive disorder.*

*P < 0.05.

### Factor Analysis and Reliability

**FIGURE 1:**
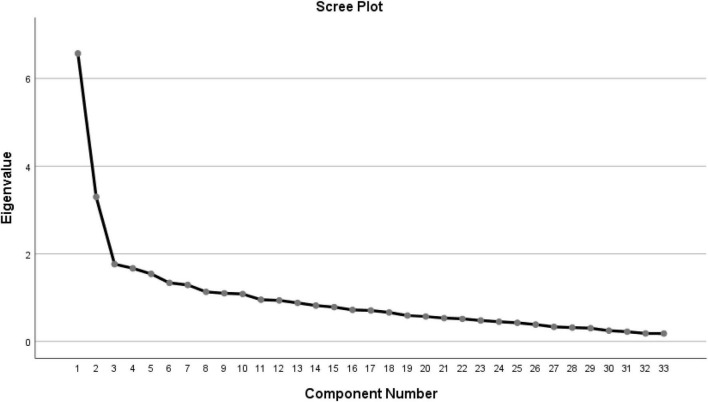
Scree plot for the HCL-33-EA.

Principal component analysis yielded 10 factors with Eigenvalues greater than 1, explaining 63.1% of the total variance (Figure 1). Factor I and Factor II had Eigenvalues of 6.6 and 3.3, respectively. Factor I consisted of 14 items (2–5, 10–12, 15, 17–19, 21, 23, 27), labeled “active/elated.” In contrast, Factor II consisted of 6 items (8–9, 14, 24–26) labeled “risk-taking/distracted.” The remaining 7 factors with Eigenvalues greater than 1 were excluded because each included 3 or fewer items, making characterization difficult. Finally, a two-factor solution was retained. The Cronbach’s alpha was 0.82 for the HCL-33-EA, 0.90 for Factor I, and 0.66 for Factor II. The Spearman-Brown Coefficient was 0.73 for the HCL-33-EA (Table 4).

**TABLE 4:** Factor loadings of the HCL-33-EA in adolescents with mood disorders (*n* = 260).

HCL-33-EA items	Active/Elated loadings	Risk-taking/Distracted factor loadings
Item 1	0.05	0.39
Item 2	**0.69**	0.13
Item 3	**0.69**	−0.11
Item 4	**0.58**	−0.10
Item 5	**0.54**	0.29
Item 6	0.24	0.31
Item 7	0.01	0.32
Item 8	0.10	**0.65**
Item 9	0.20	**0.40**
Item 10	**0.47**	0.17
Item 11	**0.69**	0.03
Item 12	**0.68**	−0.12
Item 13	0.31	0.21
Item 14	0.13	**0.57**
Item 15	**0.41**	0.21
Item 16	−0.01	0.25
Item 17	**0.78**	−0.02
Item 18	**0.76**	−0.10
Item 19	**0.62**	0.07
Item 20	−0.25	0.38
Item 21	**0.51**	0.12
Item 22	0.26	0.12
Item 23	**0.70**	−0.02
Item 24	−0.27	**0.61**
Item 25	−0.34	**0.60**
Item 26	−0.30	**0.60**
Item 27	**0.79**	−0.01
Item 28	0.003	0.11
Item 29	0.06	0.23
Item 30	−0.08	0.27
Item 31	−0.10	0.29
Item 32	−0.05	0.33
Item 33	0.18	0.35

*Bold values = loading ≥ 0.4.*

### Receiver Operating Characteristic Curve Analysis and Positive and Negative Predictive Values

**FIGURE 2:**
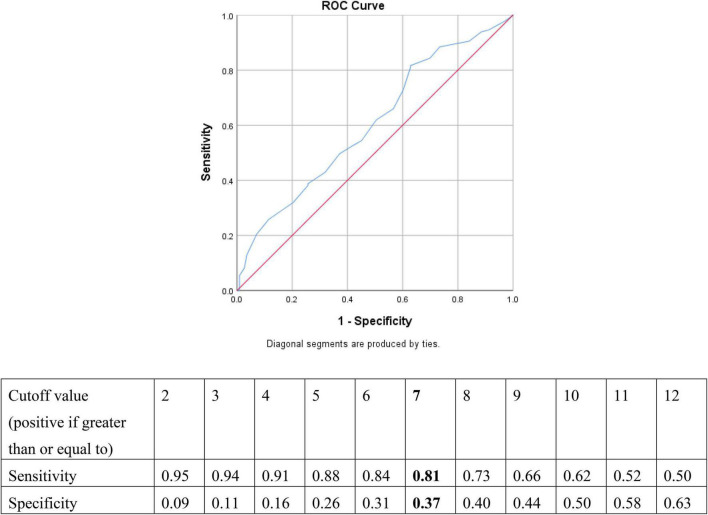
Sensitivity and specificity for each cut-off of the HCL-33-EA total score when comparing MDD and BD in adolescents.

The ROC curve analysis revealed that the HCL-33-EA total score was able to differentiate BD from MDD (*p* = 0.004), with an AUC value of 0.61 [95% confidence interval (CI): 0.54–0.67]. The optimal cut-off score of 7 generated the best combination of sensitivity (0.81) and specificity (0.37) for discriminating between the two disorders (Figure 2). The potential of the positive (PPV) and negative (NPV) predictive values of the HCL-33-EA to differentiate BD from MDD were 0.60 and 0.63, respectively with the cutoff of 7 (Figure 2).

## Discussion

To the best of our knowledge, this was the first study that validated the HCL-33-EA in depressed adolescents. The optimal cut-off score on the HCL-33-EA for distinguishing BD from MDD in adolescents was 7, which is lower than the corresponding value for the self-administered HCL-33 in depressed adolescents (11). The cut-off value on the HCL-33-EA is also lower than the corresponding figure of the HCL-32, the earlier version of this self-assessment tool. These discrepancies could be attributable to the overly sensitive identification of hypomanic symptoms by patients’ carers. Although not completely matched, the factor loadings in this study largely overlapped with the findings on the HCL-33-EA in depressed adults in China (10) and in Poland (12). The AUC value of 0.61 (95% CI: 0.54–0.67) in this study was also similar to the value reported in the HCL-33 validation study in depressed Chinese adolescents (AUC = 0.63, 95% CI: 0.55–0.71) (11).

In line with the results on the HCL-33-EA in Chinese adults with mood disorders (10) and Polish mood disorder patients (12), a two-factor structure (“active/elated” and “risk-taking/distracted”) of the HCL-33-EA was found in this study. However, the factor loadings differed from those found in earlier studies (10, 12), a discrepancy possibly due to socio-cultural influences on the expression of mood symptoms and to differences between the clinical features of adolescent and adult patients. The factor analysis of the HCL-33-EA found 13 items with factor loadings of less than 0.4, indicating that those factors may not be suitable for depressed Chinese adolescents. One example was item 7 “*tends to drive faster or take more risks when driving.*” In China, the minimum age requirement for obtaining a driving license is 18; thus, this item was not relevant to the adolescents in this study.

The HCL-33-EA had good internal consistency (Cronbach’s alpha = 0.82) and acceptable split half reliability (Spearman-Brown Coefficient = 0.73), which is consistent with the result of the validation of the self-report HCL-33 in Chinese depressed adolescents (11). The Cronbach’s alpha was 0.90 for factor I, and 0.66 for factor II. The Cronbach’s alpha was similar to the results of the HCL-33 validation study in depressed Chinese adolescents, where the Cronbach’s alpha values were 0.82, with 0.89 for factor I, and 0.67 for factor II (11).

The HCL-33-EA had a high sensitivity (0.81) and low specificity (0.37) at the optimal cut-off value of 7 in this study, a result which is probably due to the fact that the HCL-33-EA was completed by the patients’ carers. The PPV of the HCL-33-EA in this study was similar to that of the HCL-33 in depressed Chinese adolescents (0.60 vs. 0.47), while the NPV was lower (0.63 vs. 0.78) (11). The fact that the HCL-33-EA is assessed by the patients’ carers, while the HCL-33 is a self-report instrument may well account for the differences in the PPV and NPV findings between the instruments.

Several limitations of the study need to be acknowledged. First, only one hospital was included, therefore, the generalizability of the findings is likely to be limited. The HCL-33-EA should be validated in a variety of regions and clinical settings in China. Second, the use of psychotropic medications and psychiatric comorbidities were not examined, which may have biased the results to an uncertain extent. Third, the low specificity of 0.37 and the AUC value of 0.61 may be partly due to the relatively small sample size; hence, validation study on the HCL-33-EA should be conducted on a significantly larger sample size. Fourth, the test-retest reliability and the convergent/discriminant validity of the HCL-33-EA’s were not examined in the adolescents’ carers. Fifth, compared to adult patients, adolescents are more likely to present atypical features of BD, therefore, the diagnosis of BD subtypes was not made in adolescents in the participating hospital. Consequently, the psychometric properties of the HCL-33-EA could not be tested separately according to different BD subtypes. Finally, clinical manifestations of affective disorders including depressive symptoms are considerably influenced by the given socioeconomic context (19, 20), which may have biased the findings of this study to an uncertain extent.

## Conclusion

In conclusion, the Chinese version of the HCL-33-EA seems to be a useful tool for screening for BD in depressed adolescents. However, due to its high sensitivity (0.81) and low specificity (0.37) at the optimal cut-off value of 7, the HCL-33-EA needs to be further refined for adolescent patients.

## Data Availability Statement

The raw data supporting the conclusions of this article will be made available by the authors, without undue reservation.

## Ethics Statement

The Medical Ethics Committee of Beijing Anding Hospital that approved the study prohibits the authors from making the research dataset of clinical studies publicly available. Readers and all interested researchers may contact Y-TX (xyutly@gmail.com) for details. Y-TX will apply to the Medical Ethics Committee of Beijing Anding Hospital for the release of the data.

## Author Contributions

YF and Y-TX: study design. XC, HC, WB, and SS: data collection, analysis, and interpretation. HC, TC, GU, and Y-TX: drafting of the manuscript. JA: critical revision of the manuscript. All authors contributed to the article and approved the submitted version

## Conflict of Interest

The authors declare that the research was conducted in the absence of any commercial or financial relationships that could be construed as a potential conflict of interest.

## Publisher’s Note

All claims expressed in this article are solely those of the authors and do not necessarily represent those of their affiliated organizations, or those of the publisher, the editors and the reviewers. Any product that may be evaluated in this article, or claim that may be made by its manufacturer, is not guaranteed or endorsed by the publisher.

## References

[1] Joslyn C Hawes DJ Hunt C Mitchell PB. Is age of onset associated with severity, prognosis, and clinical features in bipolar disorder? A meta-analytic review. *Bipolar Disord.* (2016) 18:389–403. 10.1111/bdi.12419 27530107

[2] Baldessarini RJ Tondo L Vázquez GH Undurraga J Bolzani L Yildiz A Age at onset versus family history and clinical outcomes in 1,665 international bipolar-I disorder patients. *World Psychiatry.* (2012) 11:40–6. 10.1016/j.wpsyc.2012.01.006 22295008 PMC3266753

[3] Post RM Leverich GS Kupka RW Keck PE Jr. McElroy SL Altshuler LL Early-onset bipolar disorder and treatment delay are risk factors for poor outcome in adulthood. *J Clin Psychiatry.* (2010) 71:864–72. 10.4088/JCP.08m04994yel 20667291

[4] Van Meter AR Moreira ALR Youngstrom EA. Meta-analysis of epidemiologic studies of pediatric bipolar disorder. *J Clin Psychiatry.* (2011) 72:1250–6. 10.4088/JCP.10m06290 21672501

[5] Kowatch RA Youngstrom EA Danielyan A Findling RL. Review and meta-analysis of the phenomenology and clinical characteristics of mania in children and adolescents. *Bipolar Disord.* (2005) 7:483–96. 10.1111/j.1399-5618.2005.00261.x 16403174

[6] Van Meter AR Burke C Kowatch RA Findling RL Youngstrom EA. Ten-year updated meta-analysis of the clinical characteristics of pediatric mania and hypomania. *Bipol Disord.* (2016) 18:19–32. 10.1111/bdi.12358 26748678

[7] Culpepper L. Misdiagnosis of bipolar depression in primary care practices. *J Clin Psychiatry.* (2014) 75:5–5. 10.4088/JCP.13019tx1c 24717386

[8] Smith DJ Forty L Russell E Caesar S Walters J Cooper C Sub-threshold manic symptoms in recurrent major depressive disorder are a marker for poor outcome. *Acta Psychiatr Scand.* (2009) 119:325–9. 10.1111/j.1600-0447.2008.01324.x 19120045

[9] Feng Y Xiang Y-T Huang W Wang G Feng L Tian T-F The 33-item Hypomania Checklist (HCL-33): a new self-completed screening instrument for bipolar disorder. *J Affect Disord.* (2016) 190:214–20. 10.1016/j.jad.2015.09.057 26519642

[10] Fang M Wang Y-Y Feng Y Ungvari GS Ng CH Wang G Exploration of the psychometric properties of the 33-item hypomania checklist-external assessment (HCL-33-EA). *J Affect Disord.* (2019) 245:987–90. 10.1016/j.jad.2018.11.023 30699884

[11] Zhang Y Li W Zhang W-Y He F Pan H-P Cheung T Validation of the 33-item hypomania checklist (HCL-33) in screening adolescents with bipolar disorder. *J Affect Disord.* (2021) 281:786–91. 10.1016/j.jad.2020.11.062 33229023

[12] Lojko D Dudek D Angst J Siwek M Michalak M Rybakowski J. The 33-item Hypomania Checklist (HCL-33)—A study of the consistency between self-and external assessments in Polish bipolar patients. *Psychiatria Polska.* (2016) 50:1085–92. 10.12740/PP/66358 28211548

[13] World Health Organization. *The ICD-10 Classification of Mental and Behavioural Disorders: Clinical Descriptions and Diagnostic Guidelines.* Geneva: World Health Organization (1992).

[14] Hamilton M. A rating scale for depression. *J Neurol Neurosurg Psychiatry.* (1960) 23:56. 14399272 10.1136/jnnp.23.1.56 PMC495331

[15] Zheng Y Zhao J Phillips M Liu J Cai M Sun S Validity and reliability of the Chinese Hamilton depression rating scale. *Br J Psychiatry.* (1988) 152:660–4. 10.1192/bjp.152.5.660 3167442

[16] Johnson S Johnson R. *Conceptualising and Interpreting Reliability.* Coventry, UK: Ofqual (2009).

[17] Yang S Berdine G. The receiver operating characteristic (ROC) curve. *Southwest Respir Crit Care Chronic.* (2017) 5:34–6. 10.12746/swrccc.v5i19.391

[18] Youden WJ. Index for rating diagnostic tests. *Cancer.* (1950) 3:32–5. 10.1002/1097-0142(1950)3:1<32::AID-CNCR2820030106>3.0.CO;2-3 15405679

[19] Compton WM Conway KP Stinson FS Grant BF. Changes in the prevalence of major depression and comorbid substance use disorders in the United States between 1991-1992 and 2001-2002. *Am J Psychiatry.* (2006) 163:2141–7. 10.1176/ajp.2006.163.12.2141 17151166

[20] Kleinman A. Culture and depression. *N Engl J Med.* (2004) 351:951–3. 15342799 10.1056/NEJMp048078

